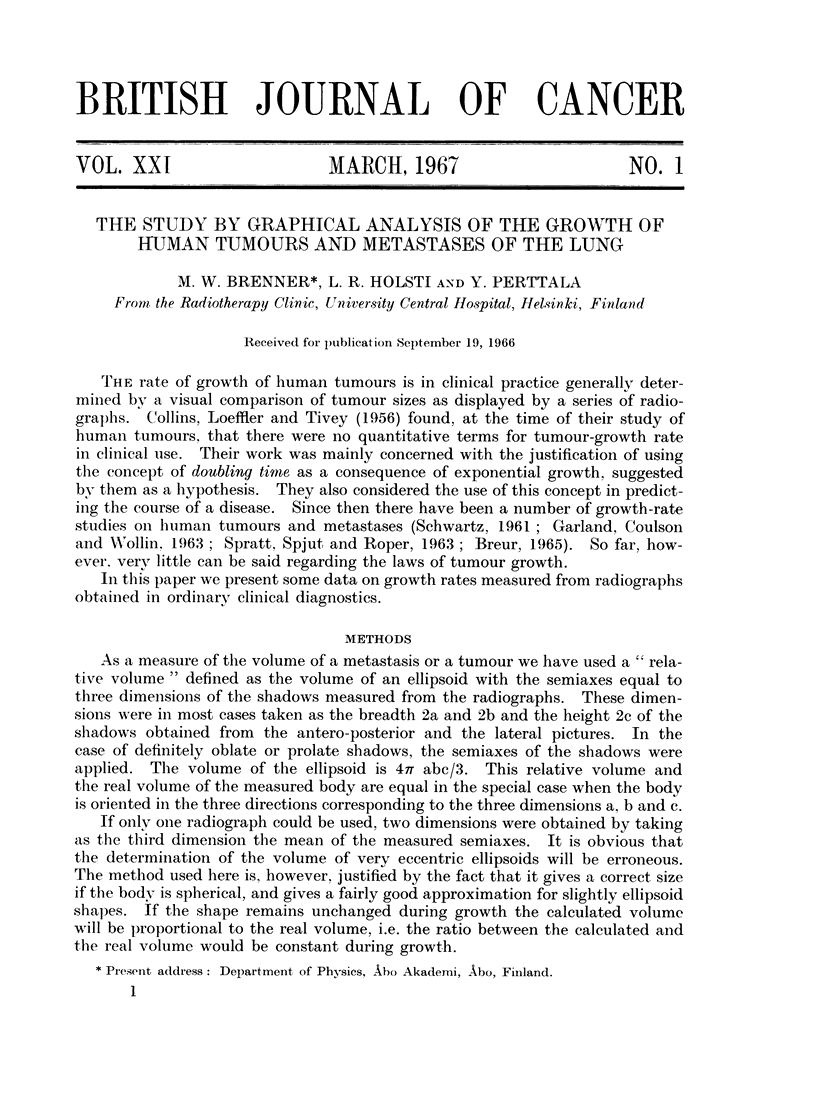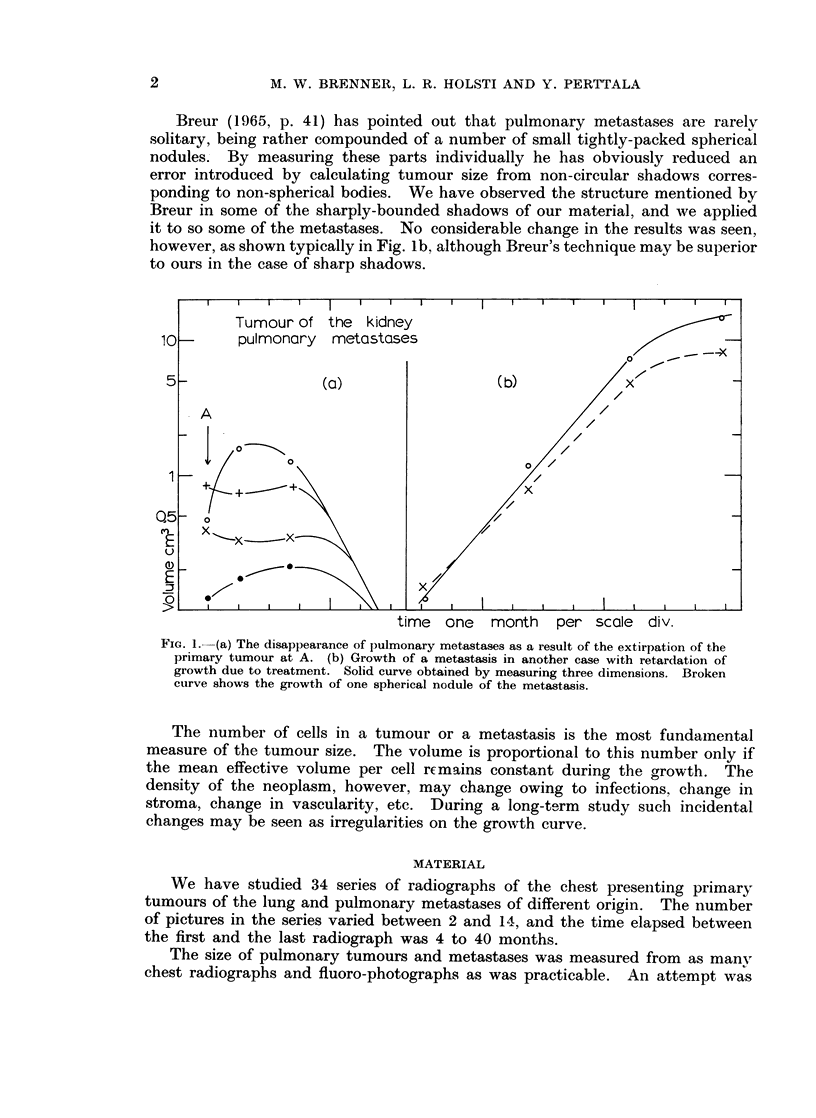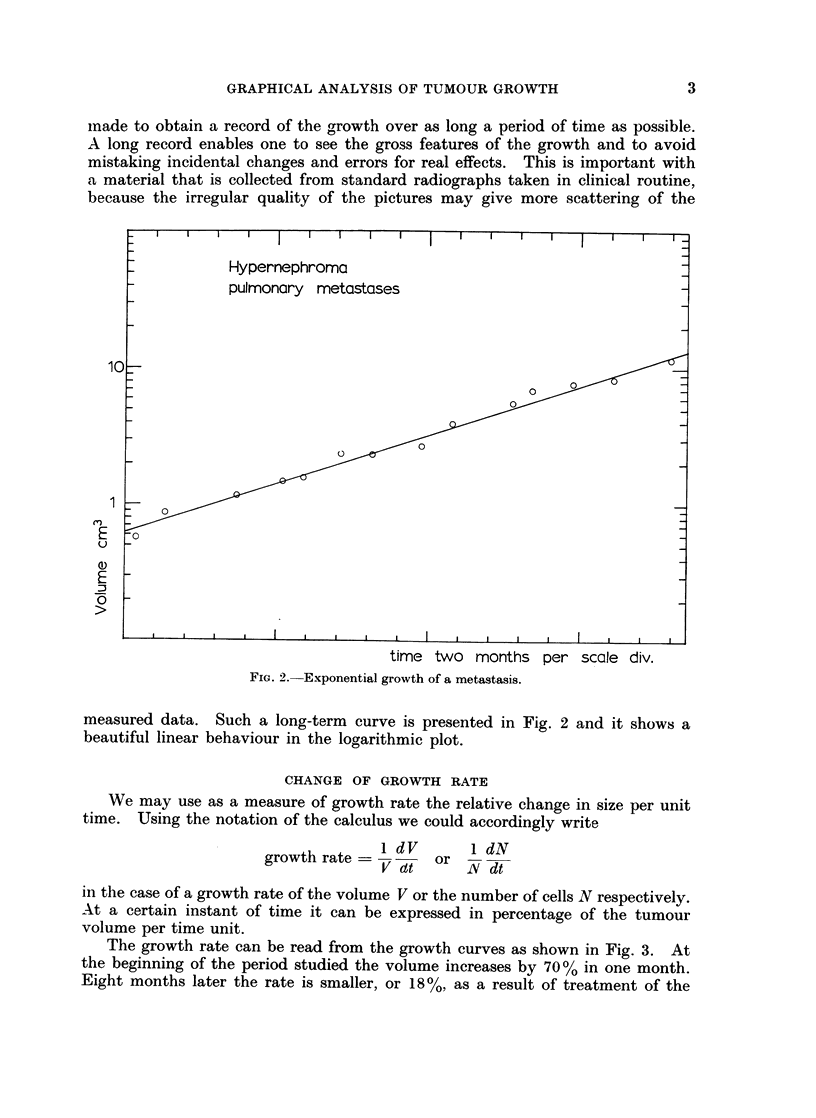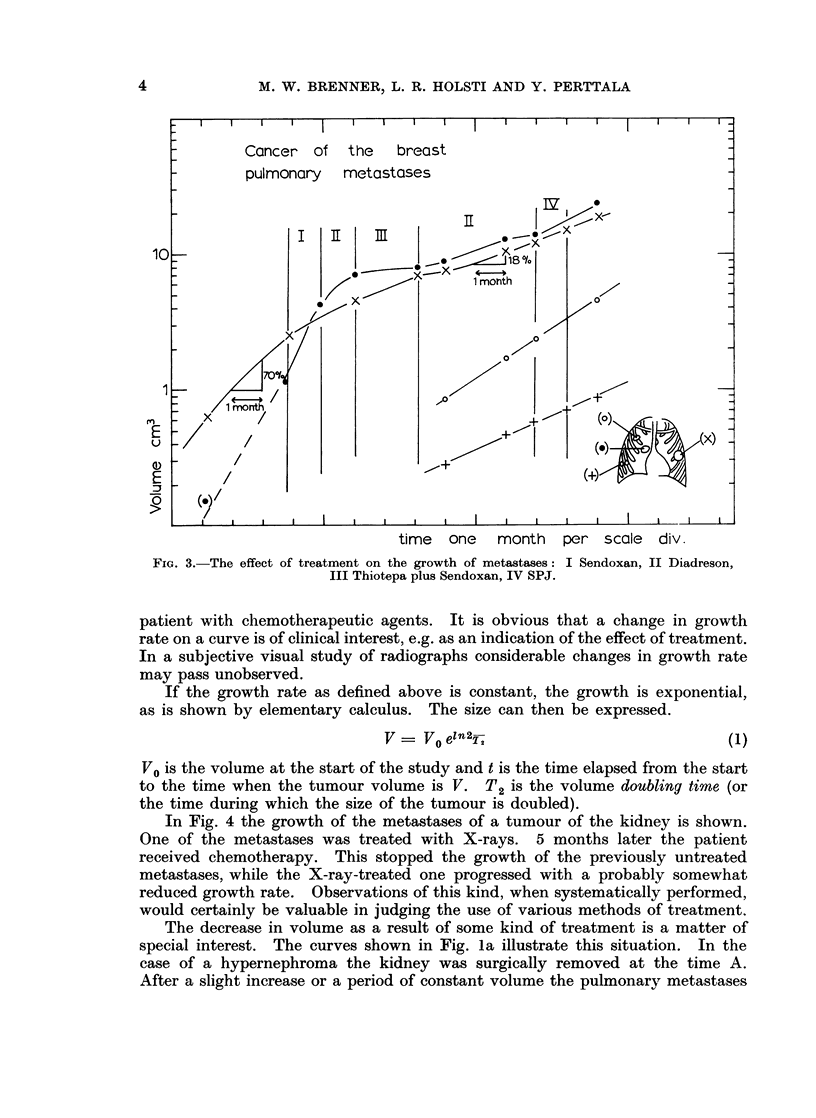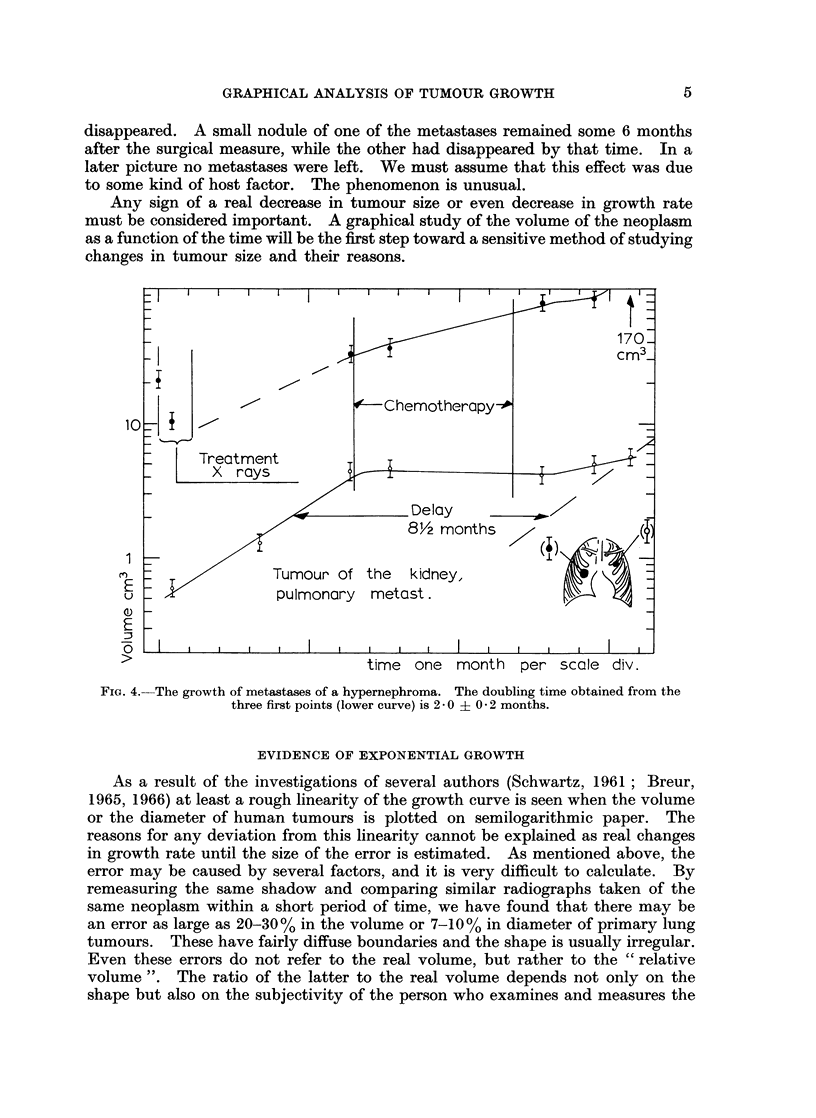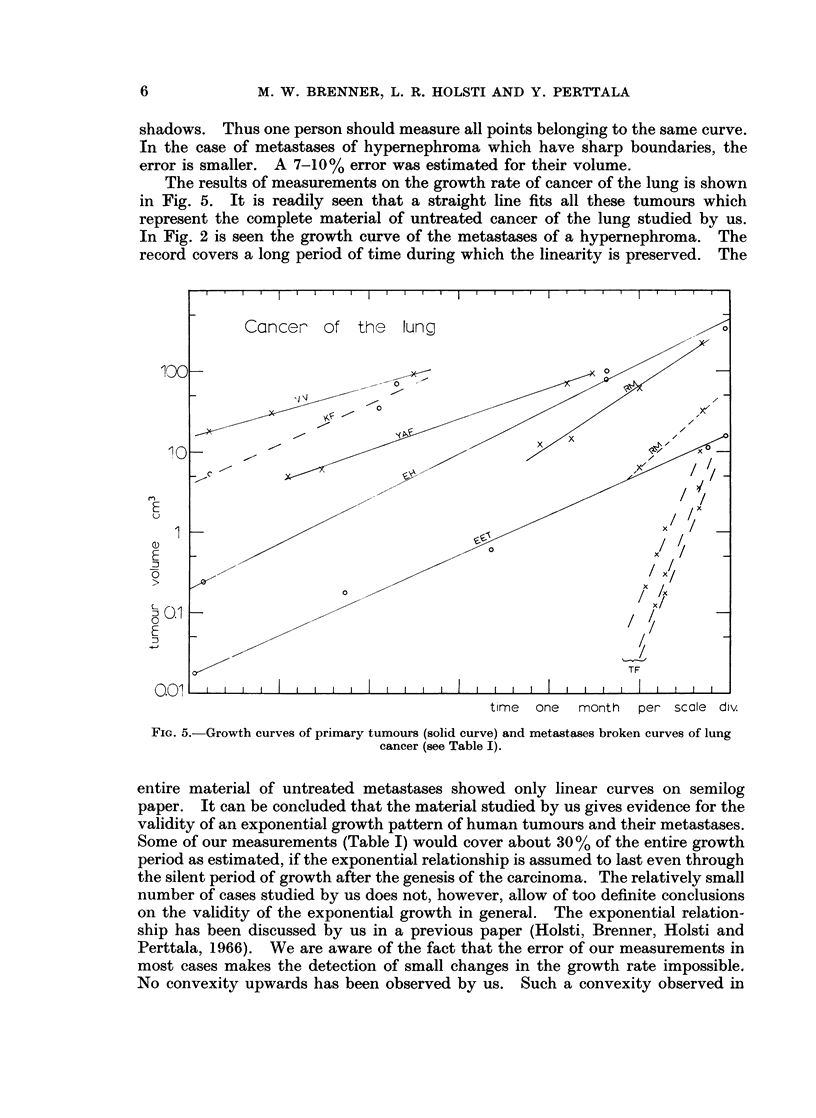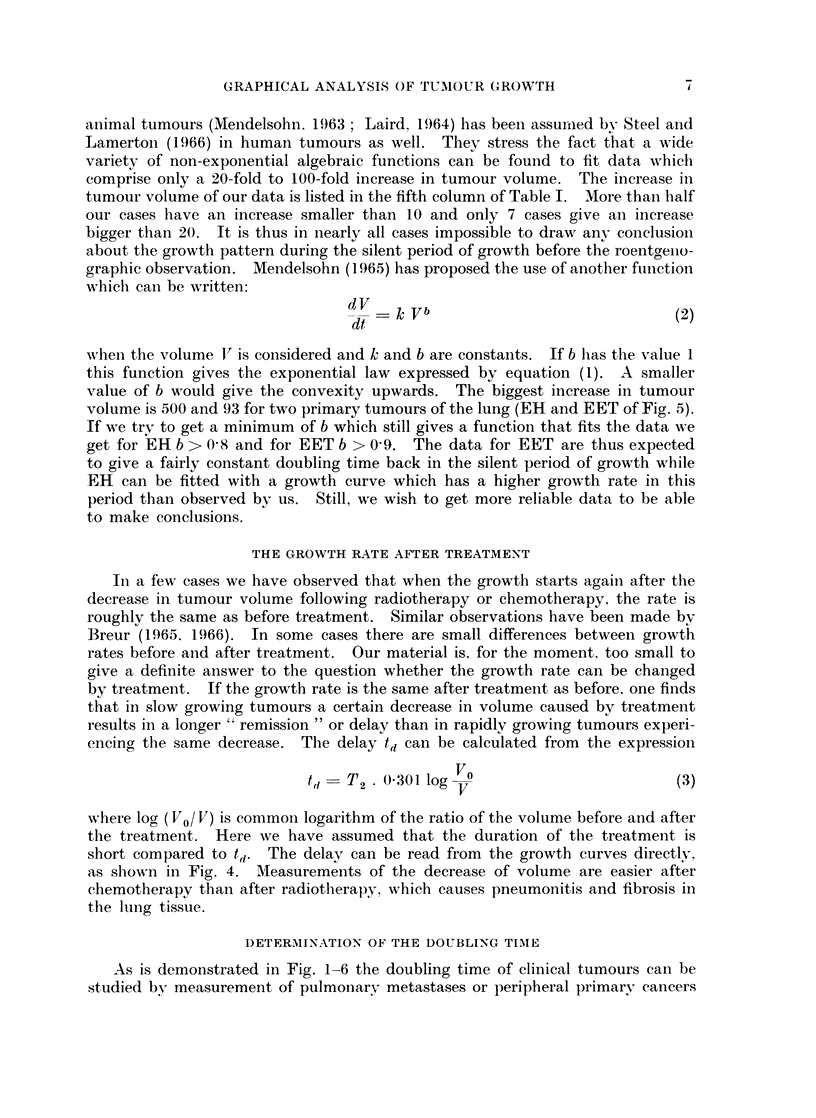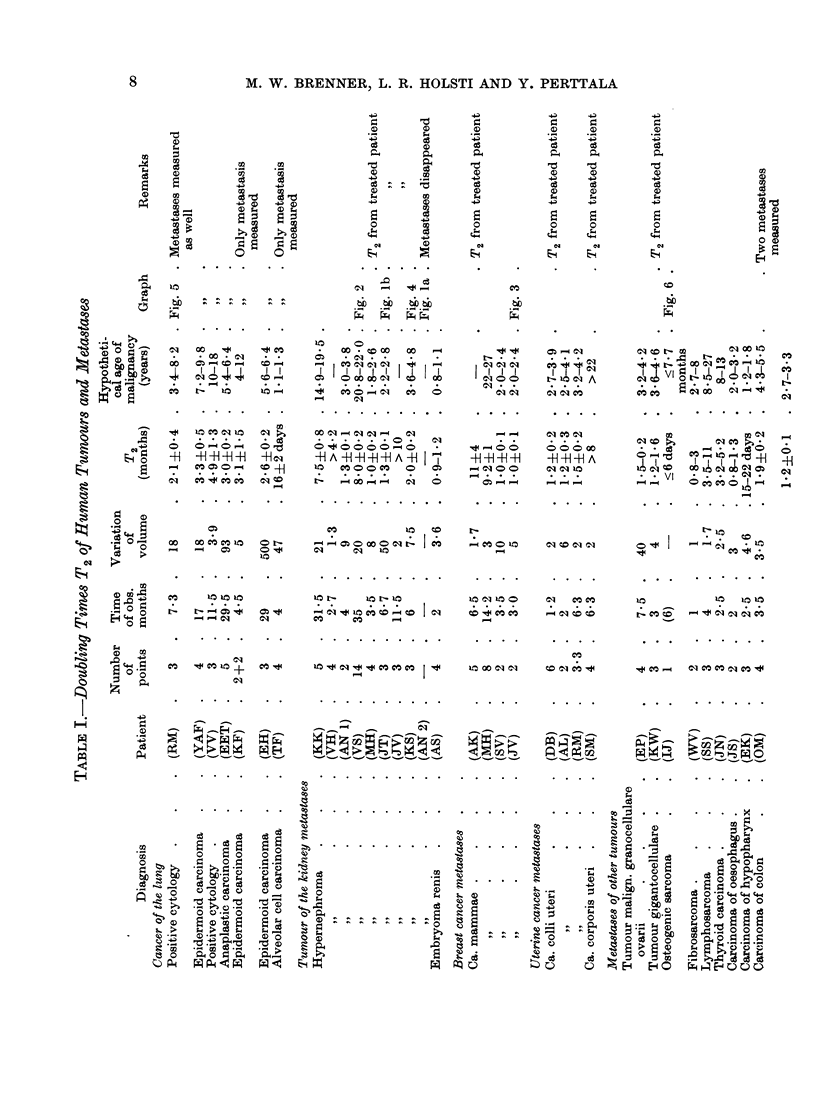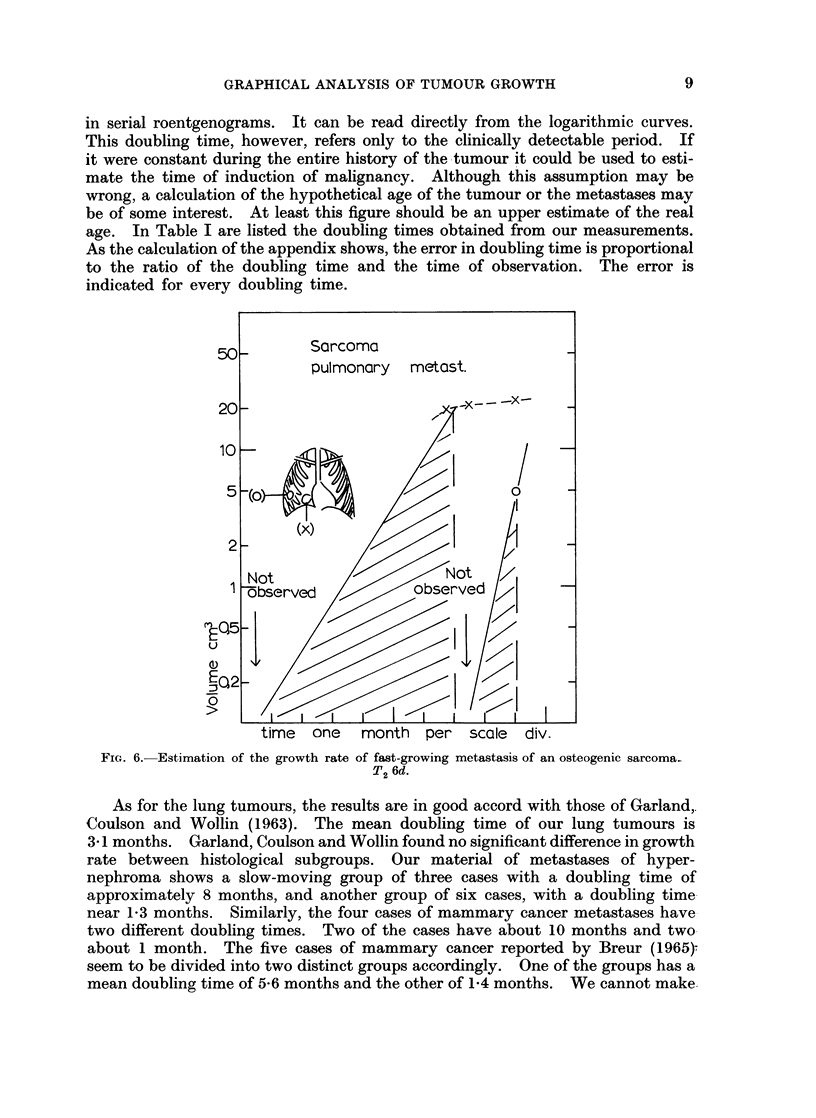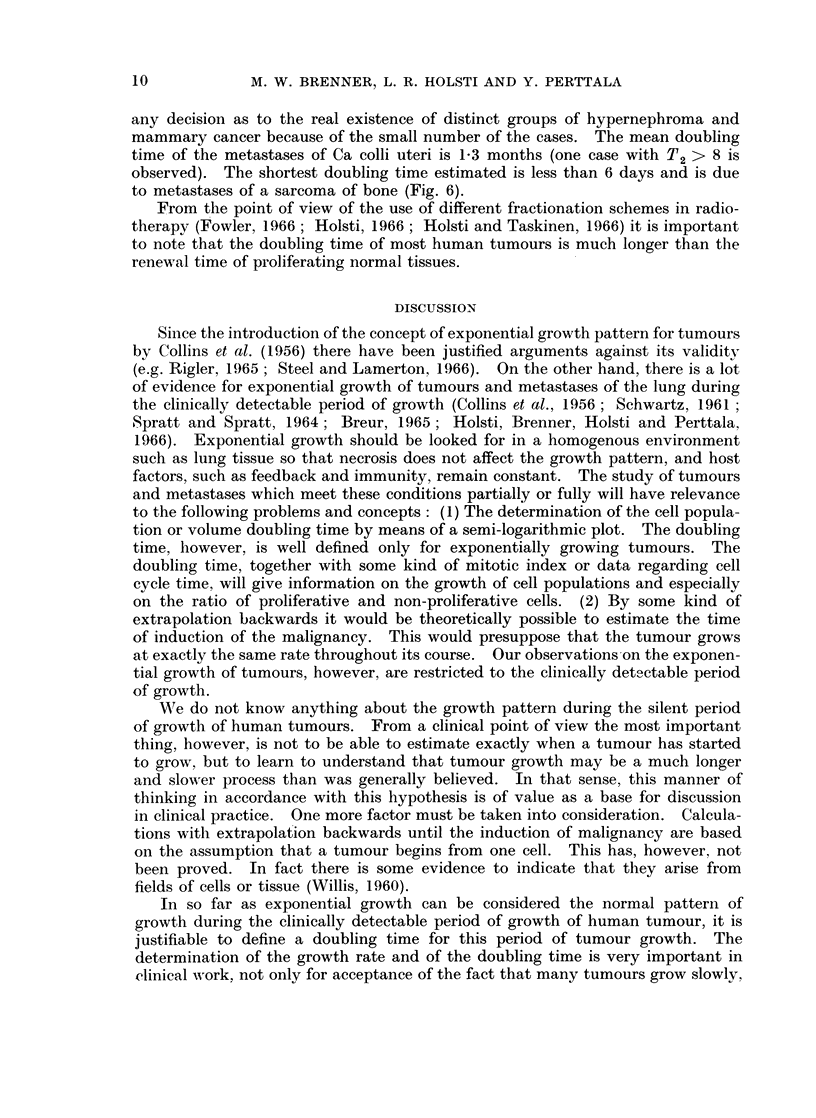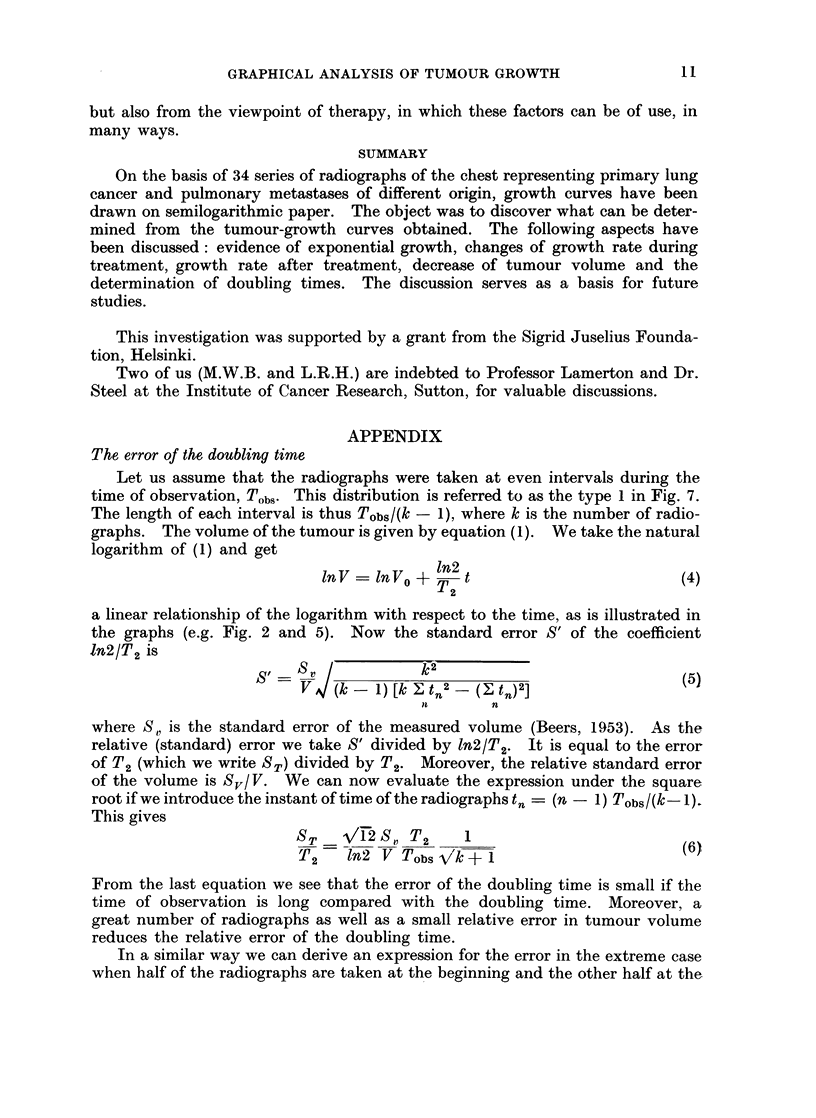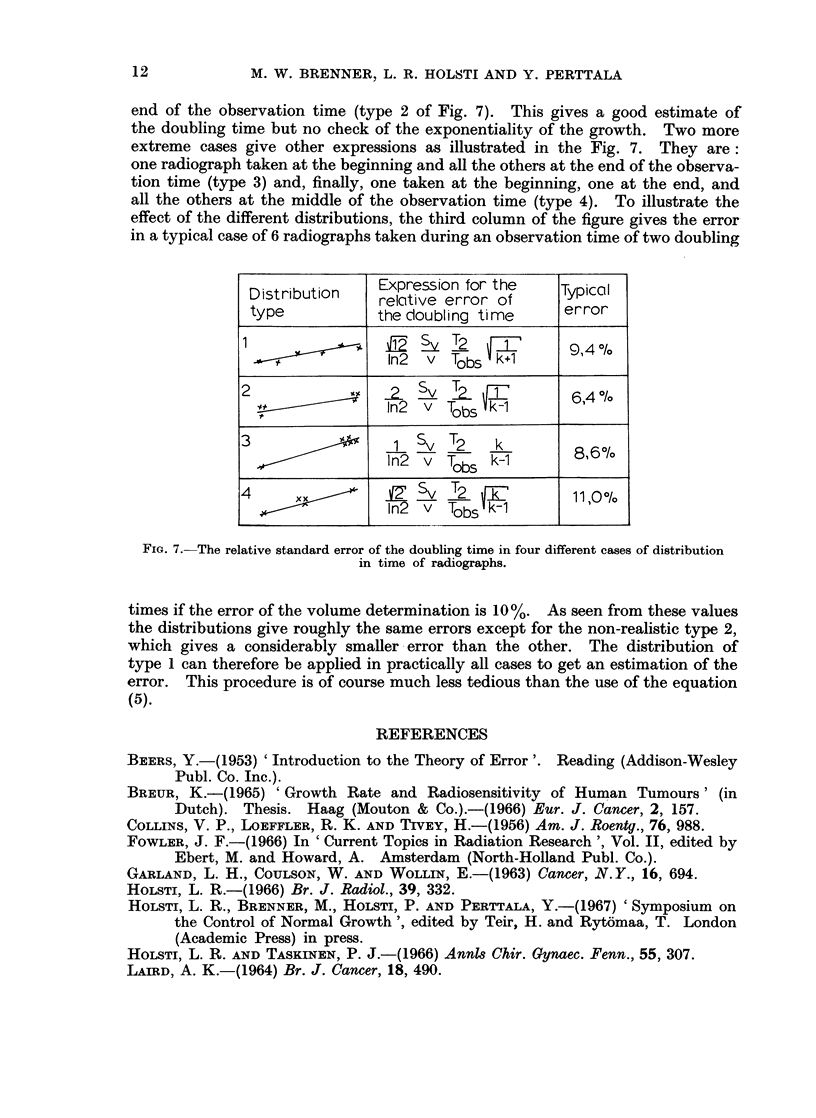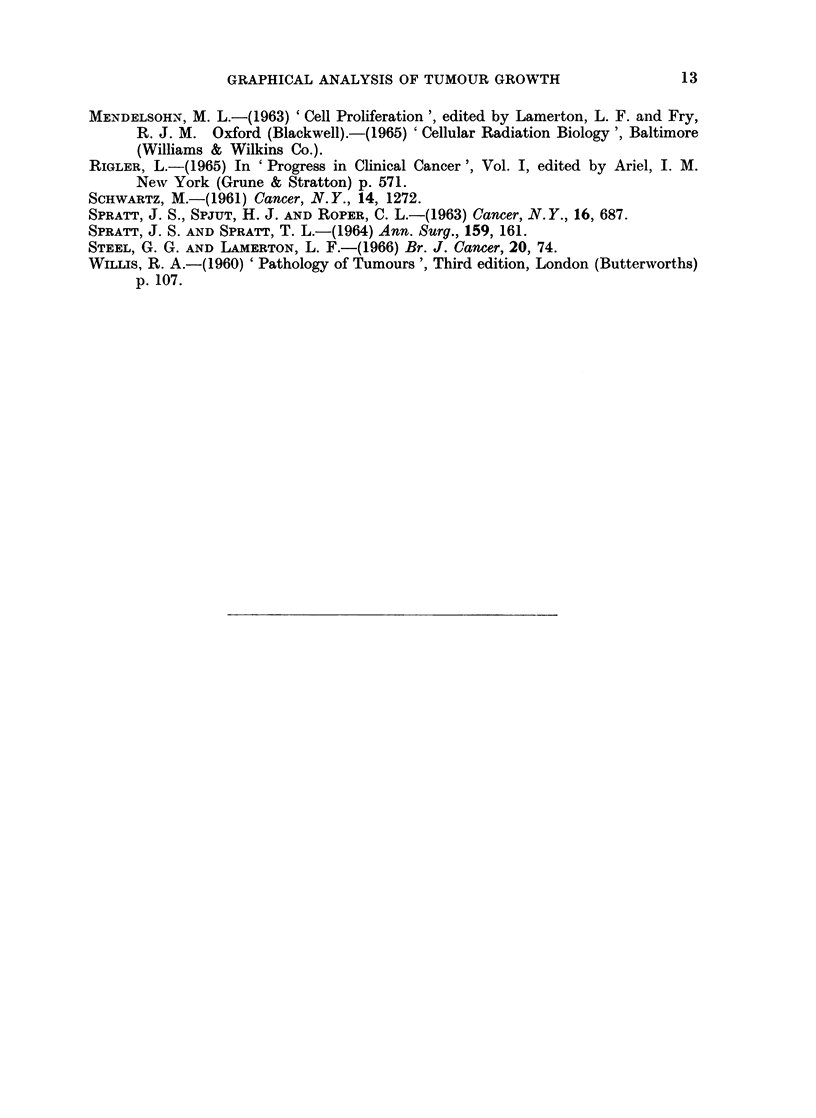# The study by graphical analysis of the growth of human tumours and metastases of the lung.

**DOI:** 10.1038/bjc.1967.1

**Published:** 1967-03

**Authors:** M. W. Brenner, L. R. Holsti, Y. Perttala


### BRITISH JOURNAL OF CANCER

VOL. XXI MARCH, 1967 NO. 1

### THE STUDY BY GRAPHICAL ANALYSIS OF THE GROWTH OF HUMAN TUMOURS AND METASTASES OF THE LUNG

M. W. BRENNER*, L. R. HOLSTI AND Y. PERTTALA

*From the Radiotherapy Clinic, University Central Hospital, Helsinki, Finland*

Received for publication September 19, 1966

THE rate of growth of human tumours is in clinical practice generally determined by a visual comparison of tumour sizes as displayed by a series of radiographs. Collins, Loeffler and Tivey (1956) found, at the time of their study of human tumours, that there were no quantitative terms for tumour-growth rate in clinical use. Their work was mainly concerned with the justification of using the concept of *doubling time* as a consequence of exponential growth, suggested by them as a hypothesis. They also considered the use of this concept in predicting the course of a disease. Since then there have been a number of growth-rate studies on human tumours and metastases (Schwartz, 1961 ; Garland, Coulson and Wollin, 1963 ; Spratt, Spjut and Roper, 1963 ; Breur, 1965). So far, however, very little can be said regarding the laws of tumour growth.

In this paper we present some data on growth rates measured from radiographs obtained in ordinary clinical diagnostics.

### METHODS

As a measure of the volume of a metastasis or a tumour we have used a " relative volume " defined as the volume of an ellipsoid with the semiaxes equal to three dimensions of the shadows measured from the radiographs. These dimensions were in most cases taken as the breadth 2a and 2b and the height 2c of the shadows obtained from the antero-posterior and the lateral pictures. In the case of definitely oblate or prolate shadows, the semiaxes of the shadows were applied. The volume of the ellipsoid is $4\pi$ abc/3. This relative volume and the real volume of the measured body are equal in the special case when the body is oriented in the three directions corresponding to the three dimensions a, b and c.

If only one radiograph could be used, two dimensions were obtained by taking as the third dimension the mean of the measured semiaxes. It is obvious that the determination of the volume of very eccentric ellipsoids will be erroneous. The method used here is, however, justified by the fact that it gives a correct size if the body is spherical, and gives a fairly good approximation for slightly ellipsoid shapes. If the shape remains unchanged during growth the calculated volume will be proportional to the real volume, i.e. the ratio between the calculated and the real volume would be constant during growth.

* Present address : Department of Physics, Åbo Akademi, Åbo, Finland.

Breur (1965, p. 41) has pointed out that pulmonary metastases are rarely solitary, being rather compounded of a number of small tightly-packed spherical nodules. By measuring these parts individually he has obviously reduced an error introduced by calculating tumour size from non-circular shadows corresponding to non-spherical bodies. We have observed the structure mentioned by Breur in some of the sharply-bounded shadows of our material, and we applied it to so some of the metastases. No considerable change in the results was seen, however, as shown typically in Fig. 1b, although Breur's technique may be superior to ours in the case of sharp shadows.

FIG. 1.—(a) The disappearance of pulmonary metastases as a result of the extirpation of the primary tumour at A. (b) Growth of a metastasis in another case with retardation of growth due to treatment. Solid curve obtained by measuring three dimensions. Broken curve shows the growth of one spherical nodule of the metastasis.

The number of cells in a tumour or a metastasis is the most fundamental measure of the tumour size. The volume is proportional to this number only if the mean effective volume per cell remains constant during the growth. The density of the neoplasm, however, may change owing to infections, change in stroma, change in vascularity, etc. During a long-term study such incidental changes may be seen as irregularities on the growth curve.

### MATERIAL

We have studied 34 series of radiographs of the chest presenting primary tumours of the lung and pulmonary metastases of different origin. The number of pictures in the series varied between 2 and 14, and the time elapsed between the first and the last radiograph was 4 to 40 months.

The size of pulmonary tumours and metastases was measured from as many chest radiographs and fluoro-photographs as was practicable. An attempt was made to obtain a record of the growth over as long a period of time as possible. A long record enables one to see the gross features of the growth and to avoid mistaking incidental changes and errors for real effects. This is important with a material that is collected from standard radiographs taken in clinical routine, because the irregular quality of the pictures may give more scattering of the

FIG. 2.—Exponential growth of a metastasis.

measured data. Such a long-term curve is presented in Fig. 2 and it shows a beautiful linear behaviour in the logarithmic plot.

### CHANGE OF GROWTH RATE

We may use as a measure of growth rate the relative change in size per unit time. Using the notation of the calculus we could accordingly write

$$\text{growth rate} = \frac{1}{V}\frac{dV}{dt} \quad \text{or} \quad \frac{1}{N}\frac{dN}{dt}$$

in the case of a growth rate of the volume $V$ or the number of cells $N$ respectively. At a certain instant of time it can be expressed in percentage of the tumour volume per time unit.

The growth rate can be read from the growth curves as shown in Fig. 3. At the beginning of the period studied the volume increases by 70% in one month. Eight months later the rate is smaller, or 18%, as a result of treatment of the

FIG. 3.—The effect of treatment on the growth of metastases: I Sendoxan, II Diadreson, III Thiotepa plus Sendoxan, IV SPJ.

patient with chemotherapeutic agents. It is obvious that a change in growth rate on a curve is of clinical interest, e.g. as an indication of the effect of treatment. In a subjective visual study of radiographs considerable changes in growth rate may pass unobserved.

If the growth rate as defined above is constant, the growth is exponential, as is shown by elementary calculus. The size can then be expressed.

$$V = V_0 e^{\ln 2 \frac{t}{T_2}} \quad (1)$$

$V_0$ is the volume at the start of the study and $t$ is the time elapsed from the start to the time when the tumour volume is $V$. $T_2$ is the volume *doubling time* (or the time during which the size of the tumour is doubled).

In Fig. 4 the growth of the metastases of a tumour of the kidney is shown. One of the metastases was treated with X-rays. 5 months later the patient received chemotherapy. This stopped the growth of the previously untreated metastases, while the X-ray-treated one progressed with a probably somewhat reduced growth rate. Observations of this kind, when systematically performed, would certainly be valuable in judging the use of various methods of treatment.

The decrease in volume as a result of some kind of treatment is a matter of special interest. The curves shown in Fig. 1a illustrate this situation. In the case of a hypernephroma the kidney was surgically removed at the time A. After a slight increase or a period of constant volume the pulmonary metastases disappeared. A small nodule of one of the metastases remained some 6 months after the surgical measure, while the other had disappeared by that time. In a later picture no metastases were left. We must assume that this effect was due to some kind of host factor. The phenomenon is unusual.

Any sign of a real decrease in tumour size or even decrease in growth rate must be considered important. A graphical study of the volume of the neoplasm as a function of the time will be the first step toward a sensitive method of studying changes in tumour size and their reasons.

FIG. 4.—The growth of metastases of a hypernephroma. The doubling time obtained from the three first points (lower curve) is $2 \cdot 0 \pm 0 \cdot 2$ months.

### EVIDENCE OF EXPONENTIAL GROWTH

As a result of the investigations of several authors (Schwartz, 1961 ; Breur, 1965, 1966) at least a rough linearity of the growth curve is seen when the volume or the diameter of human tumours is plotted on semilogarithmic paper. The reasons for any deviation from this linearity cannot be explained as real changes in growth rate until the size of the error is estimated. As mentioned above, the error may be caused by several factors, and it is very difficult to calculate. By remeasuring the same shadow and comparing similar radiographs taken of the same neoplasm within a short period of time, we have found that there may be an error as large as 20–30% in the volume or 7–10% in diameter of primary lung tumours. These have fairly diffuse boundaries and the shape is usually irregular. Even these errors do not refer to the real volume, but rather to the " relative volume ". The ratio of the latter to the real volume depends not only on the shape but also on the subjectivity of the person who examines and measures the shadows. Thus one person should measure all points belonging to the same curve. In the case of metastases of hypernephroma which have sharp boundaries, the error is smaller. A 7–10% error was estimated for their volume.

The results of measurements on the growth rate of cancer of the lung is shown in Fig. 5. It is readily seen that a straight line fits all these tumours which represent the complete material of untreated cancer of the lung studied by us. In Fig. 2 is seen the growth curve of the metastases of a hypernephroma. The record covers a long period of time during which the linearity is preserved. The

Fig. 5.—Growth curves of primary tumours (solid curve) and metastases broken curves of lung cancer (see Table I).

entire material of untreated metastases showed only linear curves on semilog paper. It can be concluded that the material studied by us gives evidence for the validity of an exponential growth pattern of human tumours and their metastases. Some of our measurements (Table I) would cover about 30% of the entire growth period as estimated, if the exponential relationship is assumed to last even through the silent period of growth after the genesis of the carcinoma. The relatively small number of cases studied by us does not, however, allow of too definite conclusions on the validity of the exponential growth in general. The exponential relationship has been discussed by us in a previous paper (Holsti, Brenner, Holsti and Perttala, 1966). We are aware of the fact that the error of our measurements in most cases makes the detection of small changes in the growth rate impossible. No convexity upwards has been observed by us. Such a convexity observed in animal tumours (Mendelsohn, 1963 ; Laird, 1964) has been assumed by Steel and Lamerton (1966) in human tumours as well. They stress the fact that a wide variety of non-exponential algebraic functions can be found to fit data which comprise only a 20-fold to 100-fold increase in tumour volume. The increase in tumour volume of our data is listed in the fifth column of Table I. More than half our cases have an increase smaller than 10 and only 7 cases give an increase bigger than 20. It is thus in nearly all cases impossible to draw any conclusion about the growth pattern during the silent period of growth before the roentgenographic observation. Mendelsohn (1965) has proposed the use of another function which can be written:

$$\frac{dV}{dt} = k\,V^b \tag{2}$$

when the volume $V$ is considered and $k$ and $b$ are constants. If $b$ has the value 1 this function gives the exponential law expressed by equation (1). A smaller value of $b$ would give the convexity upwards. The biggest increase in tumour volume is 500 and 93 for two primary tumours of the lung (EH and EET of Fig. 5). If we try to get a minimum of $b$ which still gives a function that fits the data we get for EH $b > 0{\cdot}8$ and for EET $b > 0{\cdot}9$. The data for EET are thus expected to give a fairly constant doubling time back in the silent period of growth while EH can be fitted with a growth curve which has a higher growth rate in this period than observed by us. Still, we wish to get more reliable data to be able to make conclusions.

### THE GROWTH RATE AFTER TREATMENT

In a few cases we have observed that when the growth starts again after the decrease in tumour volume following radiotherapy or chemotherapy, the rate is roughly the same as before treatment. Similar observations have been made by Breur (1965, 1966). In some cases there are small differences between growth rates before and after treatment. Our material is, for the moment, too small to give a definite answer to the question whether the growth rate can be changed by treatment. If the growth rate is the same after treatment as before, one finds that in slow growing tumours a certain decrease in volume caused by treatment results in a longer " remission " or delay than in rapidly growing tumours experiencing the same decrease. The delay $t_d$ can be calculated from the expression

$$t_d = T_2 \,.\, 0{\cdot}301 \log \frac{V_0}{V} \tag{3}$$

where log $(V_0/V)$ is common logarithm of the ratio of the volume before and after the treatment. Here we have assumed that the duration of the treatment is short compared to $t_d$. The delay can be read from the growth curves directly, as shown in Fig. 4. Measurements of the decrease of volume are easier after chemotherapy than after radiotherapy, which causes pneumonitis and fibrosis in the lung tissue.

### DETERMINATION OF THE DOUBLING TIME

As is demonstrated in Fig. 1–6 the doubling time of clinical tumours can be studied by measurement of pulmonary metastases or peripheral primary cancers

TABLE I.—*Doubling Times $T_2$ of Human Tumours and Metastases*

| Diagnosis | Patient | Number of points | Time of obs. months | Variation of volume | $T_2$ (months) | Hypothetical age of malignancy (years) | Graph | Remarks |
|---|---|---|---|---|---|---|---|---|
| *Cancer of the lung* | | | | | | | | |
| Positive cytology | (RM) | 3 | 7·3 | 18 | 2·1±0·4 | 3·4–8·2 | Fig. 5 | Metastases measured as well |
| Epidermoid carcinoma | (YAF) | 4 | 17 | 18 | 3·3±0·5 | 7·2–9·8 | ,, | |
| Positive cytology | (VV) | 3 | 11·5 | 3·9 | 4·9±1·3 | 10–18 | ,, | |
| Anaplastic carcinoma | (EET) | 5 | 29·5 | 93 | 3·0±0·2 | 5·4–6·4 | ,, | |
| Epidermoid carcinoma | (KF) | 2+2 | 4·5 | 5 | 3·1±1·5 | 4–12 | ,, | Only metastasis measured |
| Epidermoid carcinoma | (EH) | 3 | 29 | 500 | 2·6±0·2 | 5·6–6·4 | ,, | |
| Alveolar cell carcinoma | (TF) | 4 | 4 | 47 | 16±2 days | 1·1–1·3 | ,, | Only metastasis measured |
| *Tumour of the kidney metastases* | | | | | | | | |
| Hypernephroma | (KK) | 5 | 31·5 | 21 | 7·5±0·8 | 14·9–19·5 | | |
| ,, | (VH) | 4 | 2·7 | 1·3 | >4·2 | — | | |
| ,, | (AN 1) | 2 | 4 | 9 | 1·3±0·1 | 3·0–3·8 | | |
| ,, | (VS) | 14 | 35 | 20 | 8·0±0·2 | 20·8–22·0 | Fig. 2 | |
| ,, | (MH) | 4 | 3·5 | 8 | 1·0±0·2 | 1·8–2·6 | | $T_2$ from treated patient |
| ,, | (JT) | 3 | 6·7 | 50 | 1·3±0·1 | 2·2–2·8 | Fig. 1b | ,, |
| ,, | (JV) | 3 | 11·5 | 2 | >10 | — | | ,, |
| ,, | (KS) | 3 | 6 | 7·5 | 2·0±0·2 | 3·6–4·8 | Fig. 4 | |
| ,, | (AN 2) | — | — | — | — | — | Fig. 1a | Metastases disappeared |
| Embryoma renis | (AS) | 4 | 2 | 3·6 | 0·9–1·2 | 0·8–1·1 | | |
| *Breast cancer metastases* | | | | | | | | |
| Ca. mammae | (AK) | 5 | 6·5 | 1·7 | 11±4 | — | | $T_2$ from treated patient |
| ,, | (MH) | 8 | 14·2 | 3 | 9·2±1 | 22–27 | | |
| ,, | (SV) | 2 | 3·5 | 10 | 1·0±0·1 | 2·0–2·4 | | |
| ,, | (JV) | 2 | 3·0 | 5 | 1·0±0·1 | 2·0–2·4 | Fig. 3 | |
| *Uterine cancer metastases* | | | | | | | | |
| Ca. colli uteri | (DB) | 6 | 1·2 | 2 | 1·2±0·2 | 2·7–3·9 | | $T_2$ from treated patient |
| ,, | (AL) | 2 | 2 | 6 | 1·2±0·3 | 2·5–4·1 | | |
| ,, | (RM) | 3·3 | 6·3 | 2 | 1·5±0·2 | 3·2–4·2 | | |
| Ca. corporis uteri | (SM) | 4 | 6·3 | 2 | >8 | >22 | | $T_2$ from treated patient |
| *Metastases of other tumours* | | | | | | | | |
| Tumour malign. granocellulare ovarii | (EP) | 4 | 7·5 | 40 | 1·5–0·2 | 3·2–4·2 | | |
| Tumour gigantocellulare | (KW) | 3 | 3 | 4 | 1·2–1·6 | 3·6–4·6 | | $T_2$ from treated patient |
| Osteogenic sarcoma | (IJ) | 1 | (6) | — | ≤6 days | ≤7·7 months | Fig. 6 | |
| Fibrosarcoma | (WV) | 2 | 1 | 1 | 0·8–3 | 2·7–8 | | |
| Lymphosarcoma | (SS) | 3 | 4 | 1·7 | 3·5–11 | 8·5–27 | | |
| Thyroid carcinoma | (JN) | 3 | 2·5 | 2·5 | 3·2–5·2 | 8–13 | | |
| Carcinoma of oesophagus | (JS) | 2 | 2 | 3 | 0·8–1·3 | 2·0–3·2 | | |
| Carcinoma of hypopharynx | (EK) | 3 | 2·5 | 4·6 | 15–22 days | 1·2–1·8 | | |
| Carcinoma of colon | (OM) | 4 | 3·5 | 3·5 | 1·9±0·2 | 4·3–5·5 | | Two metastases measured |
| | | | | | 1·2±0·1 | 2·7–3·3 | | |

in serial roentgenograms. It can be read directly from the logarithmic curves. This doubling time, however, refers only to the clinically detectable period. If it were constant during the entire history of the tumour it could be used to estimate the time of induction of malignancy. Although this assumption may be wrong, a calculation of the hypothetical age of the tumour or the metastases may be of some interest. At least this figure should be an upper estimate of the real age. In Table I are listed the doubling times obtained from our measurements. As the calculation of the appendix shows, the error in doubling time is proportional to the ratio of the doubling time and the time of observation. The error is indicated for every doubling time.

FIG. 6.—Estimation of the growth rate of fast-growing metastasis of an osteogenic sarcoma. $T_2$ 6*d*.

As for the lung tumours, the results are in good accord with those of Garland, Coulson and Wollin (1963). The mean doubling time of our lung tumours is 3·1 months. Garland, Coulson and Wollin found no significant difference in growth rate between histological subgroups. Our material of metastases of hypernephroma shows a slow-moving group of three cases with a doubling time of approximately 8 months, and another group of six cases, with a doubling time near 1·3 months. Similarly, the four cases of mammary cancer metastases have two different doubling times. Two of the cases have about 10 months and two about 1 month. The five cases of mammary cancer reported by Breur (1965) seem to be divided into two distinct groups accordingly. One of the groups has a mean doubling time of 5·6 months and the other of 1·4 months. We cannot make any decision as to the real existence of distinct groups of hypernephroma and mammary cancer because of the small number of the cases. The mean doubling time of the metastases of Ca colli uteri is 1·3 months (one case with $T_2 > 8$ is observed). The shortest doubling time estimated is less than 6 days and is due to metastases of a sarcoma of bone (Fig. 6).

From the point of view of the use of different fractionation schemes in radiotherapy (Fowler, 1966 ; Holsti, 1966 ; Holsti and Taskinen, 1966) it is important to note that the doubling time of most human tumours is much longer than the renewal time of proliferating normal tissues.

### DISCUSSION

Since the introduction of the concept of exponential growth pattern for tumours by Collins *et al.* (1956) there have been justified arguments against its validity (e.g. Rigler, 1965 ; Steel and Lamerton, 1966). On the other hand, there is a lot of evidence for exponential growth of tumours and metastases of the lung during the clinically detectable period of growth (Collins *et al.*, 1956 ; Schwartz, 1961 ; Spratt and Spratt, 1964 ; Breur, 1965 ; Holsti, Brenner, Holsti and Perttala, 1966). Exponential growth should be looked for in a homogenous environment such as lung tissue so that necrosis does not affect the growth pattern, and host factors, such as feedback and immunity, remain constant. The study of tumours and metastases which meet these conditions partially or fully will have relevance to the following problems and concepts : (1) The determination of the cell population or volume doubling time by means of a semi-logarithmic plot. The doubling time, however, is well defined only for exponentially growing tumours. The doubling time, together with some kind of mitotic index or data regarding cell cycle time, will give information on the growth of cell populations and especially on the ratio of proliferative and non-proliferative cells. (2) By some kind of extrapolation backwards it would be theoretically possible to estimate the time of induction of the malignancy. This would presuppose that the tumour grows at exactly the same rate throughout its course. Our observations on the exponential growth of tumours, however, are restricted to the clinically detectable period of growth.

We do not know anything about the growth pattern during the silent period of growth of human tumours. From a clinical point of view the most important thing, however, is not to be able to estimate exactly when a tumour has started to grow, but to learn to understand that tumour growth may be a much longer and slower process than was generally believed. In that sense, this manner of thinking in accordance with this hypothesis is of value as a base for discussion in clinical practice. One more factor must be taken into consideration. Calculations with extrapolation backwards until the induction of malignancy are based on the assumption that a tumour begins from one cell. This has, however, not been proved. In fact there is some evidence to indicate that they arise from fields of cells or tissue (Willis, 1960).

In so far as exponential growth can be considered the normal pattern of growth during the clinically detectable period of growth of human tumour, it is justifiable to define a doubling time for this period of tumour growth. The determination of the growth rate and of the doubling time is very important in clinical work, not only for acceptance of the fact that many tumours grow slowly, but also from the viewpoint of therapy, in which these factors can be of use, in many ways.

SUMMARY

On the basis of 34 series of radiographs of the chest representing primary lung cancer and pulmonary metastases of different origin, growth curves have been drawn on semilogarithmic paper. The object was to discover what can be determined from the tumour-growth curves obtained. The following aspects have been discussed : evidence of exponential growth, changes of growth rate during treatment, growth rate after treatment, decrease of tumour volume and the determination of doubling times. The discussion serves as a basis for future studies.

This investigation was supported by a grant from the Sigrid Juselius Foundation, Helsinki.

Two of us (M.W.B. and L.R.H.) are indebted to Professor Lamerton and Dr. Steel at the Institute of Cancer Research, Sutton, for valuable discussions.

### APPENDIX

*The error of the doubling time*

Let us assume that the radiographs were taken at even intervals during the time of observation, $T_{obs}$. This distribution is referred to as the type 1 in Fig. 7. The length of each interval is thus $T_{obs}/(k - 1)$, where $k$ is the number of radiographs. The volume of the tumour is given by equation (1). We take the natural logarithm of (1) and get

$$lnV = lnV_0 + \frac{ln2}{T_2} t \tag{4}$$

a linear relationship of the logarithm with respect to the time, as is illustrated in the graphs (e.g. Fig. 2 and 5). Now the standard error $S'$ of the coefficient $ln2/T_2$ is

$$S' = \frac{S_v}{V} \sqrt{\frac{k^2}{(k-1)\,[k \sum_n t_n^2 - (\sum_n t_n)^2]}} \tag{5}$$

where $S_v$ is the standard error of the measured volume (Beers, 1953). As the relative (standard) error we take $S'$ divided by $ln2/T_2$. It is equal to the error of $T_2$ (which we write $S_T$) divided by $T_2$. Moreover, the relative standard error of the volume is $S_V/V$. We can now evaluate the expression under the square root if we introduce the instant of time of the radiographs $t_n = (n - 1)\, T_{obs}/(k-1)$. This gives

$$\frac{S_T}{T_2} = \frac{\sqrt{12}}{ln2} \frac{S_v}{V} \frac{T_2}{T_{obs}} \frac{1}{\sqrt{k+1}} \tag{6}$$

From the last equation we see that the error of the doubling time is small if the time of observation is long compared with the doubling time. Moreover, a great number of radiographs as well as a small relative error in tumour volume reduces the relative error of the doubling time.

In a similar way we can derive an expression for the error in the extreme case when half of the radiographs are taken at the beginning and the other half at the end of the observation time (type 2 of Fig. 7). This gives a good estimate of the doubling time but no check of the exponentiality of the growth. Two more extreme cases give other expressions as illustrated in the Fig. 7. They are: one radiograph taken at the beginning and all the others at the end of the observation time (type 3) and, finally, one taken at the beginning, one at the end, and all the others at the middle of the observation time (type 4). To illustrate the effect of the different distributions, the third column of the figure gives the error in a typical case of 6 radiographs taken during an observation time of two doubling

| Distribution type | Expression for the relative error of the doubling time | Typical error |
|---|---|---|
| 1  | $\frac{\sqrt{12}}{\ln 2}\,\frac{S_v}{v}\,\frac{T_2}{T_{obs}}\,\sqrt{\frac{1}{k+1}}$ | 9,4 % |
| 2  | $\frac{2}{\ln 2}\,\frac{S_v}{v}\,\frac{T_2}{T_{obs}}\,\sqrt{\frac{1}{k-1}}$ | 6,4 % |
| 3  | $\frac{1}{\ln 2}\,\frac{S_v}{v}\,\frac{T_2}{T_{obs}}\,\frac{k}{k-1}$ | 8,6% |
| 4  | $\frac{\sqrt{2}}{\ln 2}\,\frac{S_v}{v}\,\frac{T_2}{T_{obs}}\,\sqrt{\frac{k}{k-1}}$ | 11,0% |

FIG. 7.—The relative standard error of the doubling time in four different cases of distribution in time of radiographs.

times if the error of the volume determination is 10%. As seen from these values the distributions give roughly the same errors except for the non-realistic type 2, which gives a considerably smaller error than the other. The distribution of type 1 can therefore be applied in practically all cases to get an estimation of the error. This procedure is of course much less tedious than the use of the equation (5).

### REFERENCES

BEERS, Y.—(1953) 'Introduction to the Theory of Error'. Reading (Addison-Wesley Publ. Co. Inc.).

BREUR, K.—(1965) 'Growth Rate and Radiosensitivity of Human Tumours' (in Dutch). Thesis. Haag (Mouton & Co.).—(1966) *Eur. J. Cancer*, **2**, 157.

COLLINS, V. P., LOEFFLER, R. K. AND TIVEY, H.—(1956) *Am. J. Roentg.*, **76**, 988.

FOWLER, J. F.—(1966) In 'Current Topics in Radiation Research', Vol. II, edited by Ebert, M. and Howard, A. Amsterdam (North-Holland Publ. Co.).

GARLAND, L. H., COULSON, W. AND WOLLIN, E.—(1963) *Cancer, N.Y.*, **16**, 694.

HOLSTI, L. R.—(1966) *Br. J. Radiol.*, **39**, 332.

HOLSTI, L. R., BRENNER, M., HOLSTI, P. AND PERTTALA, Y.—(1967) 'Symposium on the Control of Normal Growth', edited by Teir, H. and Rytömaa, T. London (Academic Press) in press.

HOLSTI, L. R. AND TASKINEN, P. J.—(1966) *Annls Chir. Gynaec. Fenn.*, **55**, 307.

LAIRD, A. K.—(1964) *Br. J. Cancer*, **18**, 490.

MENDELSOHN, M. L.—(1963) ' Cell Proliferation ', edited by Lamerton, L. F. and Fry, R. J. M. Oxford (Blackwell).—(1965) ' Cellular Radiation Biology ', Baltimore (Williams & Wilkins Co.).
RIGLER, L.—(1965) In ' Progress in Clinical Cancer ', Vol. I, edited by Ariel, I. M. New York (Grune & Stratton) p. 571.
SCHWARTZ, M.—(1961) *Cancer, N.Y.*, **14**, 1272.
SPRATT, J. S., SPJUT, H. J. AND ROPER, C. L.—(1963) *Cancer, N.Y.*, **16**, 687.
SPRATT, J. S. AND SPRATT, T. L.—(1964) *Ann. Surg.*, **159**, 161.
STEEL, G. G. AND LAMERTON, L. F.—(1966) *Br. J. Cancer*, **20**, 74.
WILLIS, R. A.—(1960) ' Pathology of Tumours ', Third edition, London (Butterworths) p. 107.